# Mapping the aberrant brain functional connectivity in new daily persistent headache: a resting-state functional magnetic resonance imaging study

**DOI:** 10.1186/s10194-023-01577-2

**Published:** 2023-04-26

**Authors:** Wei Wang, Ziyu Yuan, Xueyan Zhang, Xiaoyan Bai, Hefei Tang, Yanliang Mei, Dong Qiu, Yingkui Zhang, Peng Zhang, Xue Zhang, Yaqing Zhang, Xueying Yu, Binbin Sui, Yonggang Wang

**Affiliations:** 1 Headache Center, Department of Neurology, Beijing Tiantan Hospital, Capital Medical University, Beijing, 100070 China; 2 Department of Neurology, The First Affiliated Hospital of Zhengzhou University, Zhengzhou, 450000 China; 3 Tiantan Neuroimaging Center of Excellence, China National Clinical Research Center for Neurological Diseases, Beijing, 100070 China; 4 Department of Radiology, Beijing Tiantan Hospital, Capital Medical University, Beijing Neurosurgical Institute, Beijing, 100070 China

**Keywords:** Functional magnetic resonance imaging, New daily persistent headache, Functional connectivity, Emotion, Pain

## Abstract

**Background and purpose:**

The pathogenesis of new daily persistent headache (NDPH) is not fully understood. We aim to map aberrant functional connectivity (FC) in patients with NDPH using resting-state functional magnetic resonance imaging (MRI).

**Methods:**

Brain structural and functional MRI data were acquired from 29 patients with NDPH and 37 well-matched healthy controls (HCs) in this cross-sectional study. Region of interest (ROI) based analysis was used to compare FC between patients and HCs, with 116 brain regions in the automated anatomical labeling (AAL) atlas were defined as seeds. The correlations between aberrant FC and patients’ clinical characteristics, and neuropsychological evaluation were also investigated.

**Results:**

Compared with HCs, patients with NDPH showed increased FC in the left inferior occipital gyrus, right thalamus and decreased FC in right lingual gyrus, left superior occipital gyrus, right middle occipital gyrus, left inferior occipital gyrus, right inferior occipital gyrus, right fusiform gyrus, left postcentral gyrus, right postcentral gyrus, right thalamus and right superior temporal gyrus. There were no correlation between FC of these brain regions and clinical characteristics, neuropsychological evaluation after Bonferroni correction (*p* > 0.05/266).

**Conclusions:**

Patients with NDPH showed aberrant FC in multiple brain regions involved in perception and regulation of emotion and pain.

**Trial registration:**

ClinicalTrials.gov Identifier: NCT05334927.

## Introduction

New daily persistent headache (NDPH) is a rare primary headache disorder with a prevalence of 0.03 to 0.1% in the general population [1]. This type of headache is characterized by an acute onset and unremitting headache that persist for > 3 months [2]. It may present as tension-type and/or migraine-like headaches with nausea, photophobia and phonophobia [3]. Although the prevalence of NDPH is rare, it is considered highly disabling owing to its persistence and therapeutic refractoriness. It often reduces patients’ quality of life and can be comorbid with sleep and psychiatric disorders, blurred vision, vertigo, lethargy and other non-specific syndromes [1, 4]. Mood disorders are more prevalent in patients with NDPH, with 65.5% having severe anxiety and 40% having severe depression [5]. To date, the pathogenesis and underlying mechanisms of NDPH are still unclear, and there is little evidence for this disease. Some reports had suggested that NDPH might be associated with viral infection, inflammatory responses, and cervical spine joint hypermobility [1, 6]. However, current etiological studies are speculative, and the factors above are common in the general population, with only a few people suffering from NDPH. Some evidence reported alterations in the structure and function of the brain in adolescents with NDPH [7]. Our previous study reported that adult patients of NDPH have decreased cerebral blood flow and arterial cerebral blood volume of multiple regions in the right hemisphere, and it provided hemodynamic evidence of NDPH [8]. However, recent evidence had demonstrated that no structural brain alterations in adult patients of NDPH, suggests that adults and adolescents with NDPH might have different brain mechanisms [9]. Neuroimaging evidence and our understanding for adult patients with NDPH remain inadequate, and its pathogenesis needs further investigation.

The functional connectivity (FC) is applied to explore the functional anatomy of neural networks and the effects of behavior, awareness, disease states, and therapeutic interventions on brain function. Numerous studies have shown that headache disorders are related to aberrant functional networks in regions involved in pain processing, perception, and emotion [10]. Investigation of FCs in patients with NDPH would be helpful to better understand the neurophysiological coupling events between different brain regions in this specific primary headache disease.

The resting-state functional magnetic resonance imaging (rs-fMRI) data in this study were obtained from patients with NDPH and healthy controls (HCs). We used seed-based rs-fMRI analysis to map FC patterns between seed voxels and the whole brain voxels and to explore aberrant FC in patients with NDPH and their correlation with clinical characteristics and neuropsychological evaluation. We also aimed to explore neuroimaging markers of NDPH and provide evidence for its pathological mechanisms. We hypothesized that: (1) adult patients with NDPH had abnormal FC in brain regions involved in perception, processing, and regulation of emotion and pain; (2) there was a significant correlation between FC of these brain regions and clinical features.

## Materials and methods

### Standard protocol approvals, registrations, and patient consent

This study used a cross-sectional, case–control design to calculate FC in patients with NDPH and HCs from rs-fMRI data. All participants were informed of the trial’s details and signed informed consent. This was a sub-study of the ongoing China HeadAche DIsorders RegiStry Study (CHAIRS, unique identifier: NCT05334927), and ethical approval was granted by the Beijing Tiantan Hospital, Capital Medical University (number: KY2022-044).

### Participants

From October 2020 to October 2022, 40 HCs and 31 patients who were diagnosed NDPH were enrolled in the Headache Center, Department of Neurology, Beijing Tiantan Hospital, Capital Medical University. The diagnosis of NDPH was based on the International Classification of Headache Diseases, 3rd edition (ICHD-3) diagnostic criteria [2]. The inclusion criteria for patients with NDPH were as follows. (1) Patients who satisfied the definition of NDPH according to the ICHD-3 criteria. The diagnostic criteria were as follows: A) a persistent headache fulfilling criteria B and C; B) headache with a distinctly remembered onset, with pain becoming persistent and unremitting within 24 h; C) a headache last for > 3 months; and D) without better explanation by another ICHD-3 diagnosis; (2) feasibility of MRI scan (participants without claustrophobic syndrome and metal implant etc.); (3) none of the patients enrolled had been prophylactically treated for NDPH for at least 3 months, and the patients with NDPH had no history of excessive use of acute treatment drugs, and (4) availability of complete data. The exclusion criteria for patients were as follows: (1) there were other headache-related secondary factors before the scan; (2) patients combined with other types of primary headache; (3) pregnancy or breastfeeding, comorbidities with other neurological, cardio-cerebrovascular, endocrine system disease, etc.; and (4) poor quality of MRI data. The inclusion criteria for HCs were (1) feasibility of MRI scan (participants without claustrophobic syndrome and metal implant etc.); (2) no neurological or other major systemic diseases; (3) demographics of HCs and patients with NDPH were matched; (4) first degree relative without headaches. The exclusion criteria for HCs were (1) pregnancy or breastfeeding, (2) MRI contraindications, and (3) poor quality of MRI data.

### Demographic data and neuropsychological tests

We recorded demographic data, including sex, age, body mass index (BMI), and the following clinical characteristics of patients with NDPH, including age at onset, disease duration, pain intensity of NDPH, and related neuropsychological test results. The pain intensity of NDPH was assessed by the visual analogue scale (VAS) (headache intensity on a 0–10 numerical rating scale). The impact degree of the headache was measured by the Headache Impact Test-6 (HIT-6) [11]. The sleep quality was assessed by Pittsburgh Sleep Quality Index (PSQI) [12], and the cognitive level was evaluated by Montreal Cognitive Assessment (MoCA) [13]. The level of depression and anxiety were assessed by Patient Health Questionnaire-9 (PHQ-9) [14] and Generalized Anxiety Disorder-7 (GAD-7) [15], respectively. The PHQ-9 score of 10 was used as the cut-off for having symptom of depression [14]. The score of GAD-7 ≥ 10 indicates having symptom of anxiety [15]. The Chinese version of the PSQI defines poor quality of sleep as an overall PSQI score > 7 [16]. The score of MoCA < 26 indicates cognitive impairment [13].

### Imaging protocols

MRI was performed by applying a 3.0 Tesla MR scanner (Signa Premier, GE Healthcare, Chicago, IL, USA) using a 48-channel head coil at the National Neurological Centre of Beijing Tiantan Hospital. Participants were required to avoid head and neck movements as much as possible, stay awake, relax, and eyes closed during MRI scanning. Using earplugs and foam padding to reduce scanner and head-movement noise. T1-weighted volumetric images were obtained by the MP-RAGE sequence with 1-mm isotropic resolution (sagittal acquisition, the field of view (FOV) = 256 mm, acquisition matrix = 256, slice number = 192, flip angle = 8°, preparation time = 880 ms, recovery time = 400 ms, acceleration factor = 2*,* acquisition time = 4:00). Rs-fMRI data were recorded by a multi-band blood-oxygenation-level-dependent (BOLD) sequence with 2.4-mm isotropic resolution (transverse acquisition, FOV = 208 mm, acquisition matrix = 86, slice number = 65 with multi-band factor = 6, flip angle = 64°, echo time/repetition time = 39/1000 ms, no in-plane acceleration or slice gap, 330 brain volumes).

### Data processing and analysis

All MRI data were processed in MATLAB 2016b using Statistical Parametric Mapping 12 (SPM12) and Data Processing Assistant for rs-fMRI Advanced Edition (DPARSFA) (MathWorks, Natick, MA, USA; http://www.rfmri.org/DPARSF). The data preprocessing steps were as follows: (1) to reduce the impact of unstable magnetization, the first 20 time points of each functional time course were eliminated; (2) slice timing; (3) headache motion correction; (4) spatial normalization; and (5) spatial smoothing (full width at half maximum = 8 mm). These steps were performed using SPM12 (FIL, London, UK). Participants with head movements exceeding 3 mm or a maximum rotation exceeding 2° in any direction were excluded. Then, the co-registered anatomical images were segmented into white matter, gray matter, and cerebrospinal fluid and spatially normalized into the standard Montreal Neurological Institute (MNI) space with a resampled voxel size of 3 × 3 × 3 mm^3^. DPARSFA was used for linear trend removal and time bandpass filtering (0.01–0.08 Hz) to reduce the influence of low-frequency drift and high-frequency noise.

The FC analysis steps were as follows. (1) We defined 116 brain regions in the automated anatomical labeling (AAL) atlas as seeds [17]. (2) FC computation of 116 brain regions in the AAL atlas were performed using SPM12. We extracted the time course of both seeds and calculated FC between the seed’s time course and the averaged time courses of the whole brain in a voxel-wise manner using Pearson’s correlation analysis. (3) To improve normality, we switched the correlation coefficients to z-values using Fisher r to z transformation. (4) The two-sample t-test for voxel level with sex and age as covariates was performed in the brain mask. The threshold was *p* < 0.001, with two-tailed (voxel level) and family wise errors (FWEs), corrected to *p* < 0.05 (cluster level). (5) The average FC values of individual significant clusters were extracted for Pearson’s correlation analysis with demographic information and neuropsychological tests.

### Statistical analyses

The sample size was based on the available data and previous literature [7]. A sample size of 66 cases (37 HC group and 29 NDPH group) would provide 90% power to reject the null hypothesis equal means when the mean difference is 0.1384 (0.4817–0.3433) with standard deviations of 0.1494 for the HC group and 0.1299 for NDPH group at a two-sided alpha of 0.05 (Power Analysis and Sample Size Software (2021). NCSS, LLC. Kaysville, Utah, USA, ncss.com/software/pass). Mean ± standard deviation and median with interquartile range were described as the normally and non-normally distributed data, respectively. The chi-square test was used to compare sex between the patient and HC groups. The independent samples t-test was applied to compare normally distributed data between the groups. The Mann–Whitney U test was applied to compare non-normally distributed data between the groups. The correlations between demographic data, neuropsychological test results, and ROI-related FC values were calculated using Pearson’s correlation analysis. Statistical significance was set at *p* < 0.05, and all hypothesis tests were two-tailed. All statistical data were analyzed by Statistical Product Service Solutions (SPSS) software for Windows (version 25.0; IBM Corp., Armonk, NY, USA). The two-sample t-test for voxel level with gender and age as covariates was performed in spm12 to evaluate the differences of FC between patients and HCs. The multiple regression model was used for voxel-based correlation analysis (age and sex were used as covariates and potentially as factors), *p* < 0.05 were considered a significant difference, and FWE correction was performed (P_FWE_ < 0.05 was considered a significant difference). Multiple corrections were applied when correlation analysis between the FC and clinical characteristics, and neuropsychological tests (Bonferroni correction, *p* = 0.00019 [0.05/266]) were conducted.

## Results

### Demographics and clinical characteristics

Thirty-one patients with NDPH and 40 HCs were prospectively recruited. Two patients with NDPH (incomplete scans, *n* = 1, excessive head movements (> 3 mm), or maximum rotation exceeding 2° in any direction, *n* = 1) and 3 HCs (incomplete scans, *n* = 1, excessive head movements (> 3 mm), or maximum rotation exceeding 2° in any direction, *n* = 2) were excluded. In total, 29 patients with NDPH (15 males, 14 females; age [mean ± SD] 37.00 ± 21.06 years old) and 37 HCs (16 males, 21 females; age [mean ± SD] 34.89 ± 10.96 years old) were included in this study. The demographic information and clinical characteristics of the participants were presented in Table 1. Nevertheless, there were no significant differences in demographic information including age (*p* = 0.627), sex (*p* = 0.497), BMI (*p* = 0.077), and right-handers (*p* > 0.999) between the patients and HCs. Four patients did not have HIT-6, PHQ-9, GAD-7, and PSQI scores and 11 patients did not have MoCA scores due to their reluctance to be assessed by the questionnaires during hospitalization and lost to follow-up after discharge, who were excluded from the analysis associated with scales. Among patients with NDPH, 16 (64.0%) had severe headache effects (HIT-6 score ≥ 56), 11 (44.0%) had depression (PHQ-9 score ≥ 10), 8 (32.0%) had generalized anxiety disorder (GAD-7 score ≥ 10), 16 (64.0%) had poor sleep quality (PSQI score > 7) and 7 (38.9%) had cognitive impairment (MoCA score < 26). No abnormalities were found on the structural MRI scans of all the participants.

**Table 1:** Participants’ demographics and clinical characteristics

	**Healthy Controls (*n* = 37)**	**NDPH (*n* = 29)**	***p*-value**
Age, years	34.89 ± 10.96	37.00 ± 21.06	0.627
Sex (male/female)	16/21 (43.24/56.76%)	15/14 (51.72/48.28%)	0.497
BMI (kg/m^2^)	22.44 ± 3.04	24.08 ± 4.06	0.077
Right-handers, n (%)	37 (100%)	29 (100%)	> 0.999
Headache laterality, n (%)			
Unilateral	NA	4 (13.79%)	NA
Bilateral	NA	25 (86.21%)	NA
Location of headache, n (%)			
Frontal region	NA	12 (41.38%)	NA
Temporal region	NA	14 (48.28%)	NA
Parietal region	NA	15 (51.72%)	NA
Occipital region	NA	11 (37.93%)	NA
Periorbital region	NA	1 (3.45%)	NA
Nausea, vomiting, n (%)	NA	4 (13.79%)	NA
Photophobia, n (%)	NA	11 (37.93%)	NA
Phonophobia, n (%)	NA	15 (51.72%)	NA
Age at onset, years	NA	17.00 (13.00, 42.00)	NA
Disease duration, years	NA	3.00 (1.00, 14.00)	NA
VAS score (0–10)	NA	4.00 (3.00, 7.00)	NA
HIT-6 score (36–78)	NA	64.64 ± 10.94	NA
PHQ-9 score (0–27)	NA	9.92 ± 6.78	NA
GAD-7 score (0–21)	NA	6.89 ± 5.17	NA
PSQI score (0–21)	NA	10.20 ± 5.03	NA
MoCA score (0–30)	NA	24.67 ± 4.64	NA

*NDPH* new daily persistent headache, *NA* not applicable, *BMI* body mass index, *VAS* Visual Analogue Scale, *HIT-6* Headache Impact Test-6, *PHQ-9* Patient Health Questionnaire-9, *GAD-7* Generalized Anxiety Disorder-7, *PSQI* Pittsburgh Sleep Quality Index, *MoCA* Montreal Cognitive Assessment

### Seed-based FC between patients and HCs

**Fig. 1:**
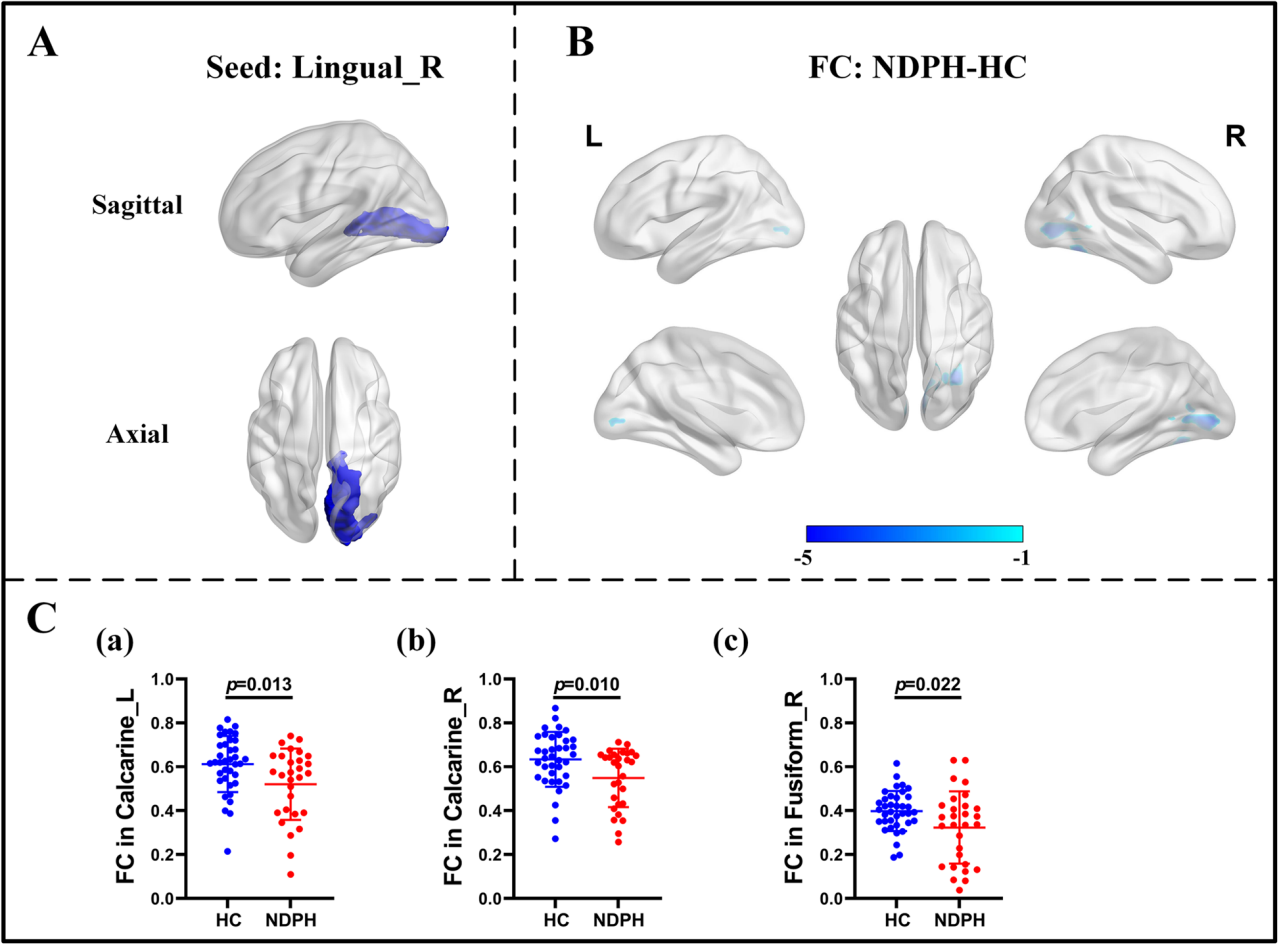
FC analysis from right lingual gyrus to the whole brain voxels in NDPH and HC groups. **A** The schematic diagram of the seed (right lingual gyrus). The brain region marked in blue represents the right lingual gyrus. **B** The schematic diagram of significant FC differences. Compared with HCs, patients with NDPH show significantly decreased FC between the seed and bilateral calcarine, right fusiform gyrus. **C** (a), (b) and (c) represent FC values between the right lingual gyrus and bilateral calcarine, right fusiform gyrus respectively in HC group and patients with NDPH. Notes: The color bar showed t-values of the two-sample t-tests on FC. Abbreviations: L, left; R, right; FC, functional connectivity; NDPH, new daily persistent headache; HC, health control

**Fig. 2:**
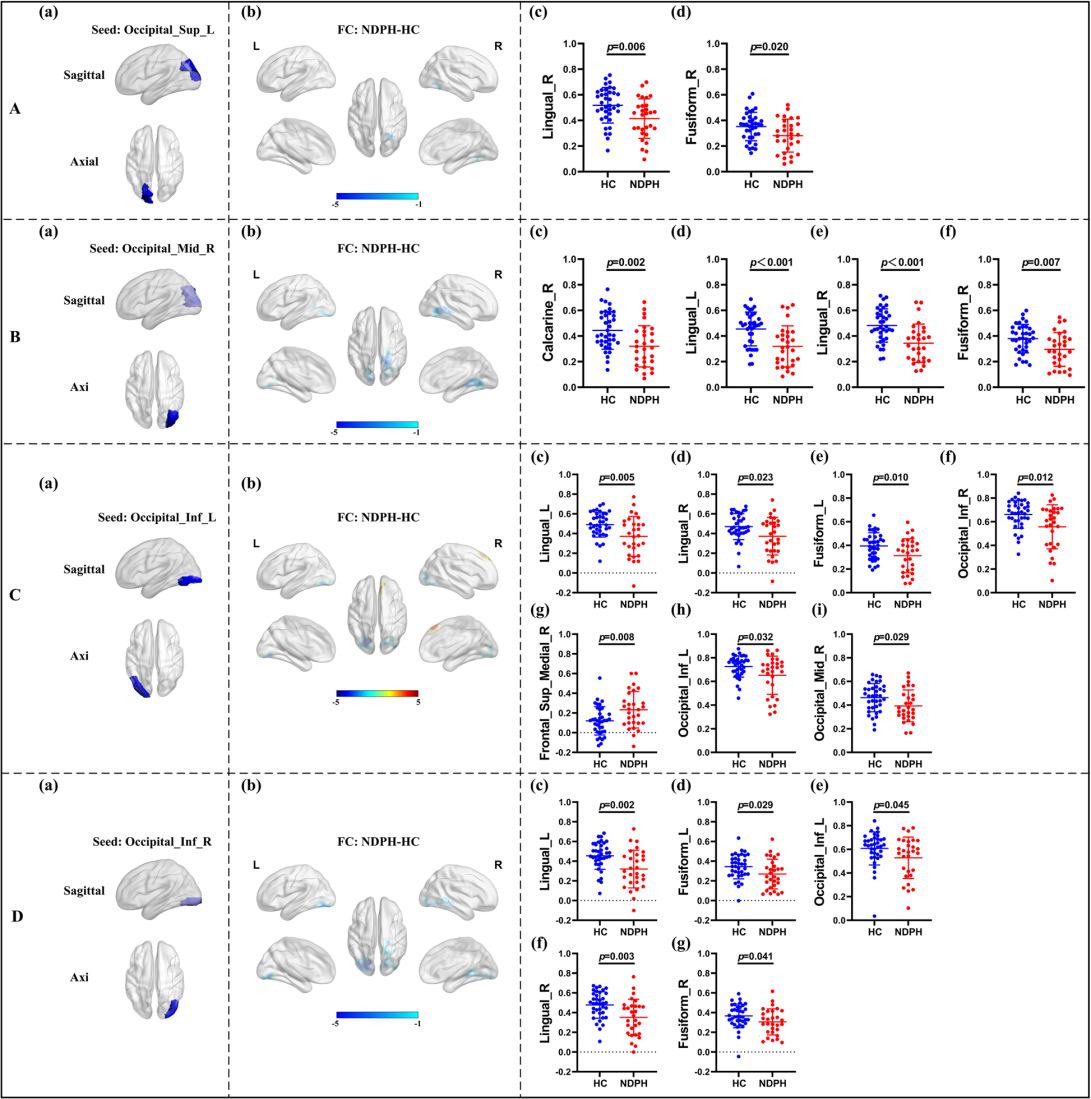
FC analysis from occipital lobe to the whole brain voxels in NDPH and HC groups. **A** (a) The schematic diagram of the seed (left superior occipital gyrus). (b) Compared with HCs, patients with NDPH showed significantly decreased FC between the left superior occipital gyrus and right lingual gyrus, right fusiform gyrus. (c) and (d) represented FC values between the seed and right lingual gyrus, right fusiform gyrus respectively in HCs and patients with NDPH.** B** (a) The schematic diagram of the seed (right middle occipital gyrus). (b) Compared with HCs, patients with NDPH showed significantly decreased FC between the seed and bilateral lingual gyrus, right calcarine, right fusiform gyrus. (c) to (f) represented FC values between the seed and bilateral lingual gyrus, right calcarine, right fusiform gyrus respectively in HCs and patients with NDPH. **C** (a) The schematic diagram of the seed (left inferior occipital gyrus). (b) Compared with HCs, patients with NDPH showed significantly decreased FC between the seed and bilateral lingual gyrus, left fusiform gyrus, bilateral inferior occipital gyrus, right middle occipital gyrus and increased FC between the seed and medial superior frontal gyrus. (c) to (i) represented FC values between the seed and bilateral lingual gyrus, left fusiform gyrus, bilateral inferior occipital gyrus, right middle occipital gyrus, medial superior frontal gyrus respectively in HCs and patients with NDPH. **D** (a) The schematic diagram of the seed (right inferior occipital gyrus). (b) Compared with HCs, patients with NDPH showed significantly decreased FC between the seed and bilateral lingual gyrus, bilateral fusiform gyrus, left inferior occipital gyrus. (c) to (g) represented FC values between the seed and bilateral lingual gyrus, bilateral fusiform gyrus, left inferior occipital gyrus respectively in HC group and patients with NDPH. Notes: The color bar showed t-values of the two-sample t-tests on FC. Abbreviations: L, left; R, right; FC, functional connectivity; Sup, superior; Mid, middle; Inf, inferior; NDPH, new daily persistent headache; HC, health control

**Fig. 3:**
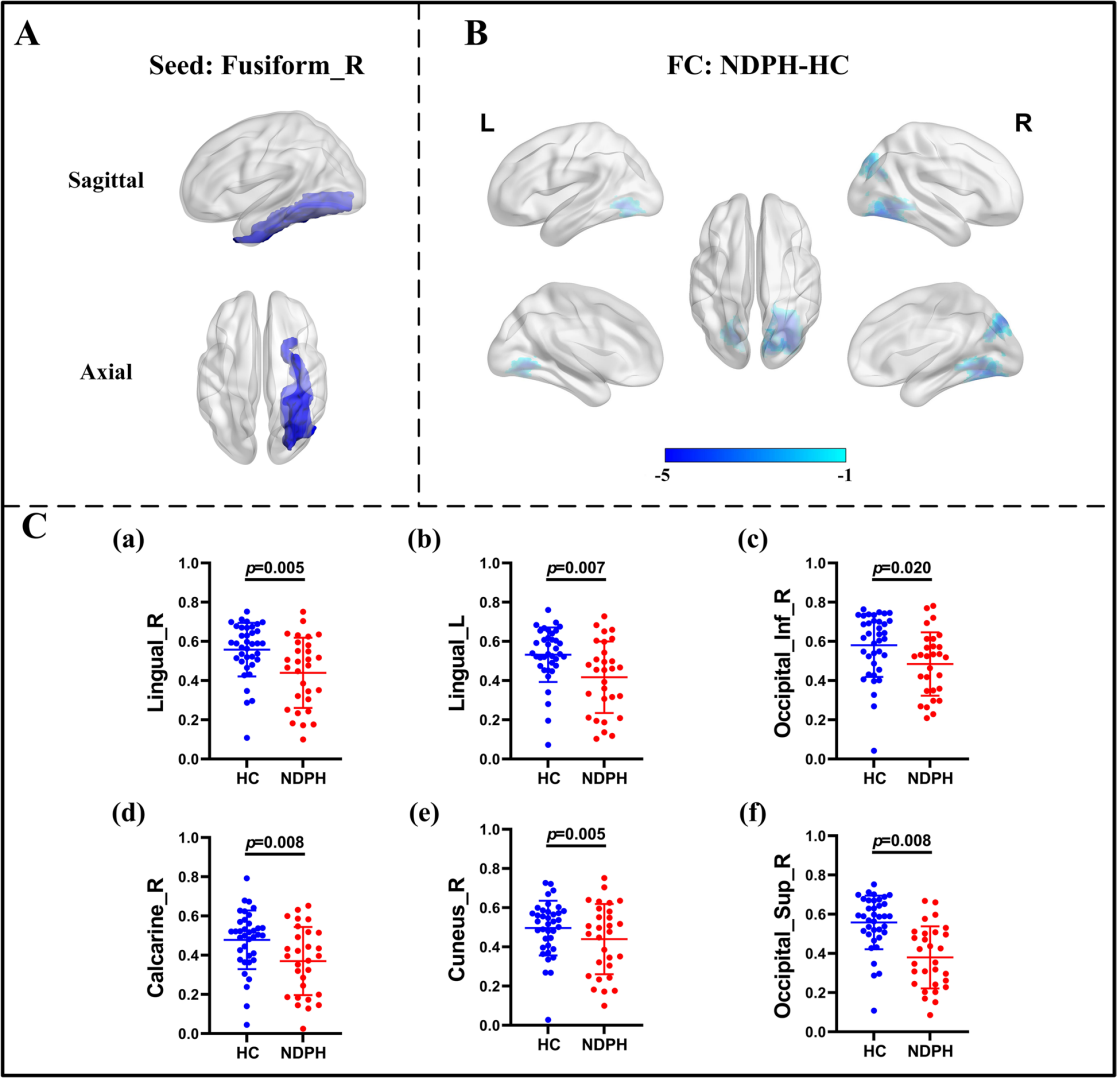
FC analysis from right fusiform gyrus to the whole brain voxels in NDPH and HC groups. **A** The schematic diagram of the seed (right fusiform gyrus). The brain region marked in blue represents the right fusiform gyrus. **B** The schematic diagram of significant FC differences. Compared with HCs, patients with NDPH showed significantly decreased FC between the seed and bilateral lingual gyrus, right inferior occipital gyrus, right calcarine, right cuneus, right superior occipital gyrus. **C** (a) to (f) represented FC values between the seed and bilateral lingual gyrus, right inferior occipital gyrus, right calcarine, right cuneus, right superior occipital gyrus respectively in HC group and patients with NDPH. Notes: The color bar showed t-values of the two-sample t-tests on FC. Abbreviations: L, left; R, right; Sup, superior; Inf, inferior; FC, functional connectivity; NDPH, new daily persistent headache; HC, health control

**Fig. 4:**
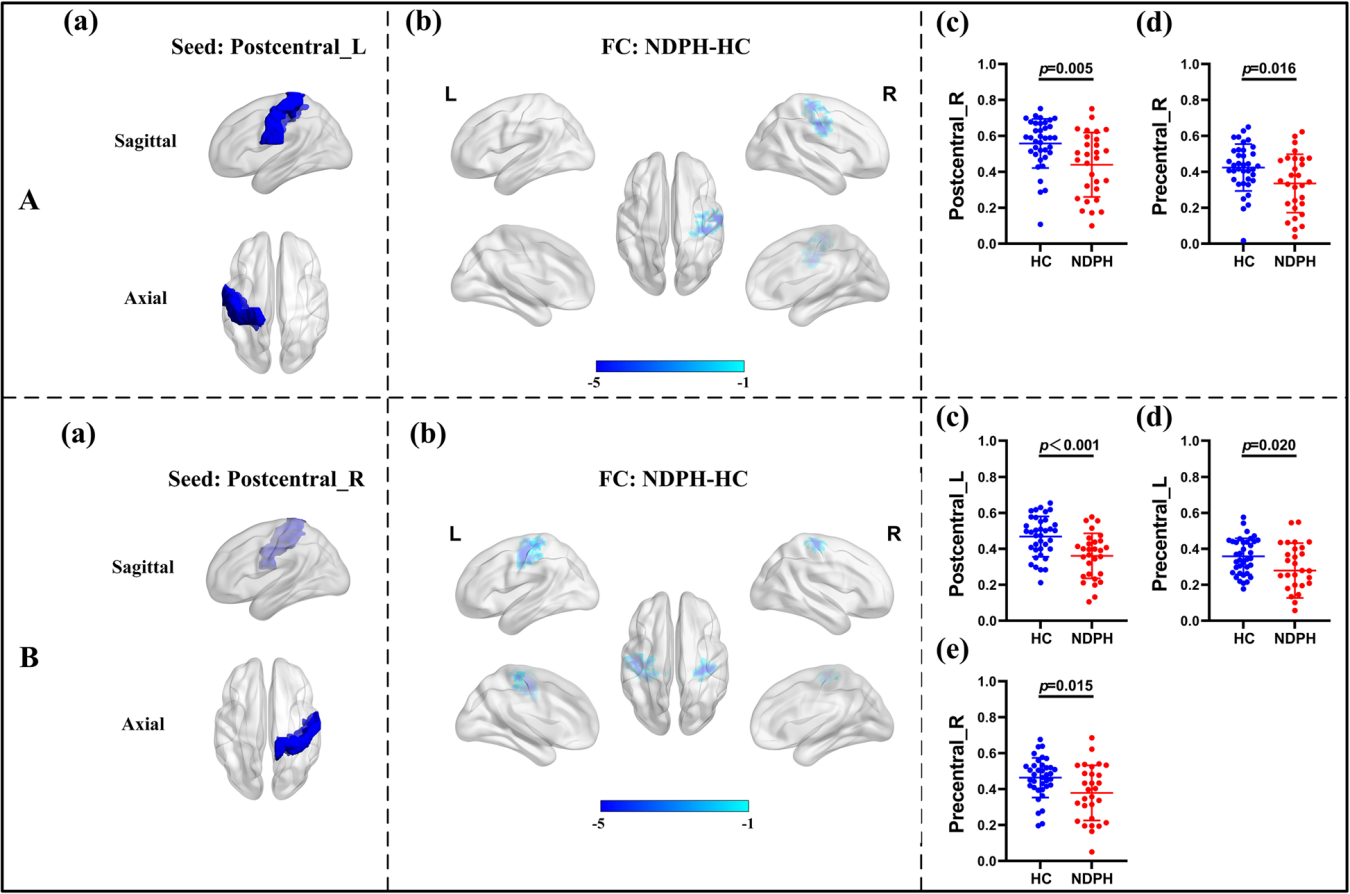
FC analysis from bilateral postcentral gyrus to the whole brain voxels in NDPH and HC groups. **A** (a) The schematic diagram of the seed (left postcentral gyrus). (b) Compared with HCs, patients with NDPH showed significantly decreased FC between the seed and right postcentral gyrus, right precentral gyrus. (c) and (d) represented FC values between the seed and right postcentral gyrus, right precentral gyrus respectively in HC group and patients with NDPH. **B** (a) The schematic diagram of the seed (right postcentral gyrus). (b) Compared with HCs, patients with NDPH showed significantly decreased FC between the seed and bilateral precentral gyrus and left postcentral gyrus. (c) to (e) represented FC values between the seed and bilateral precentral gyrus and left postcentral gyrus respectively in HC group and patients with NDPH. Notes: The color bar showed t-values of the two-sample t-tests on FC. Abbreviations: L, left; R, right; FC, functional connectivity; NDPH, new daily persistent headache; HC, health control

**Fig. 5:**
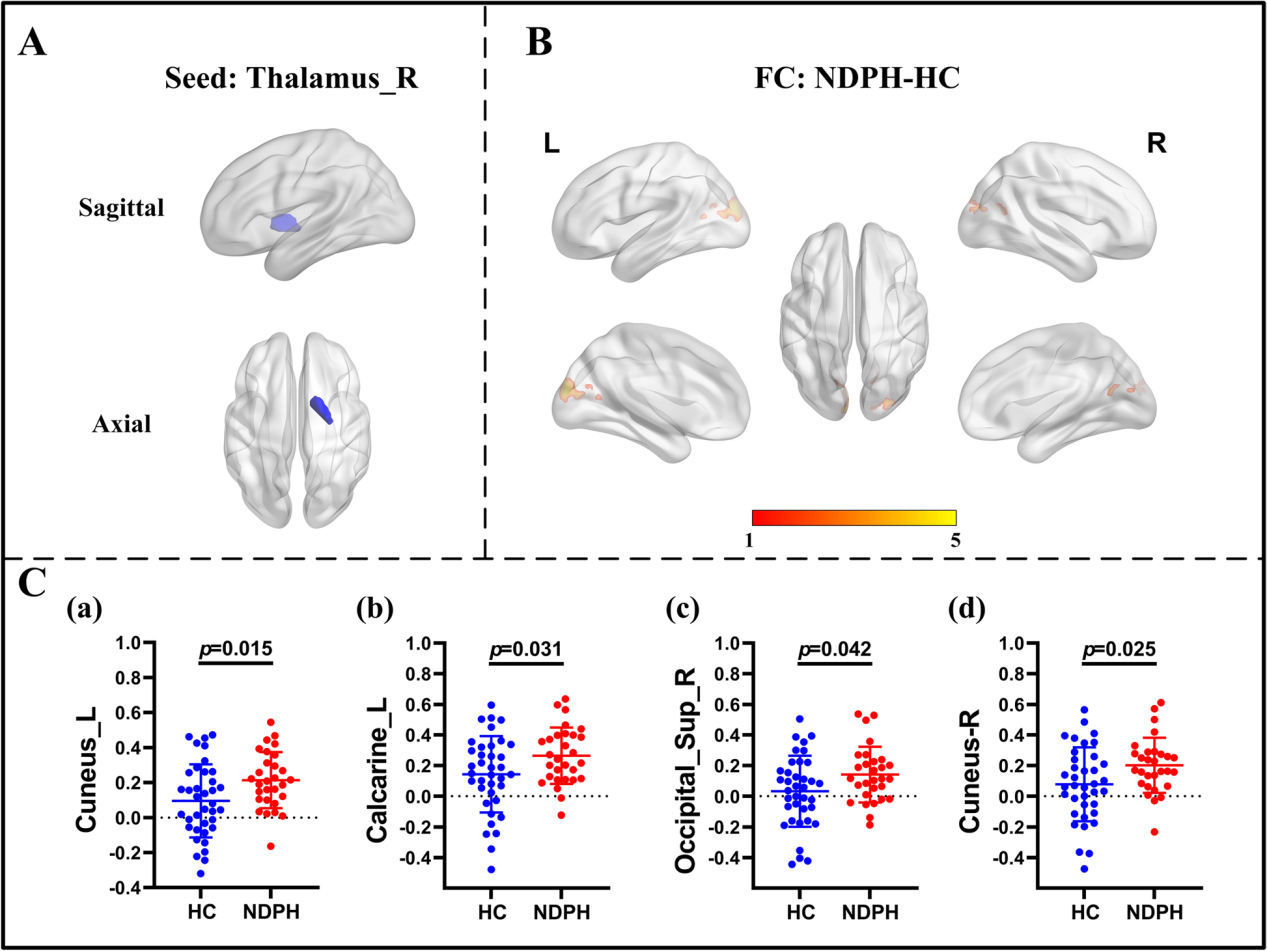
FC analysis from right thalamus to the whole brain voxels in NDPH and HC groups. **A** The schematic diagram of the seed (right thalamus). The brain region marked in blue represents the right fusiform gyrus. **B** The schematic diagram of significant FC differences. Compared with HCs, patients with NDPH showed significantly increased FC between the seed and bilateral cuneus, left calcarine, right superior occipital gyrus. **C** (a) to (d) represent FC values between the seed and bilateral cuneus, left calcarine, right superior occipital gyrus respectively in HCs and patients with NDPH. Notes: The color bar showed t-values of the two-sample t-tests on FC. Abbreviations: L, left; R, right; Sup, superior; FC, functional connectivity; NDPH, new daily persistent headache; HC, health control

**Fig. 6:**
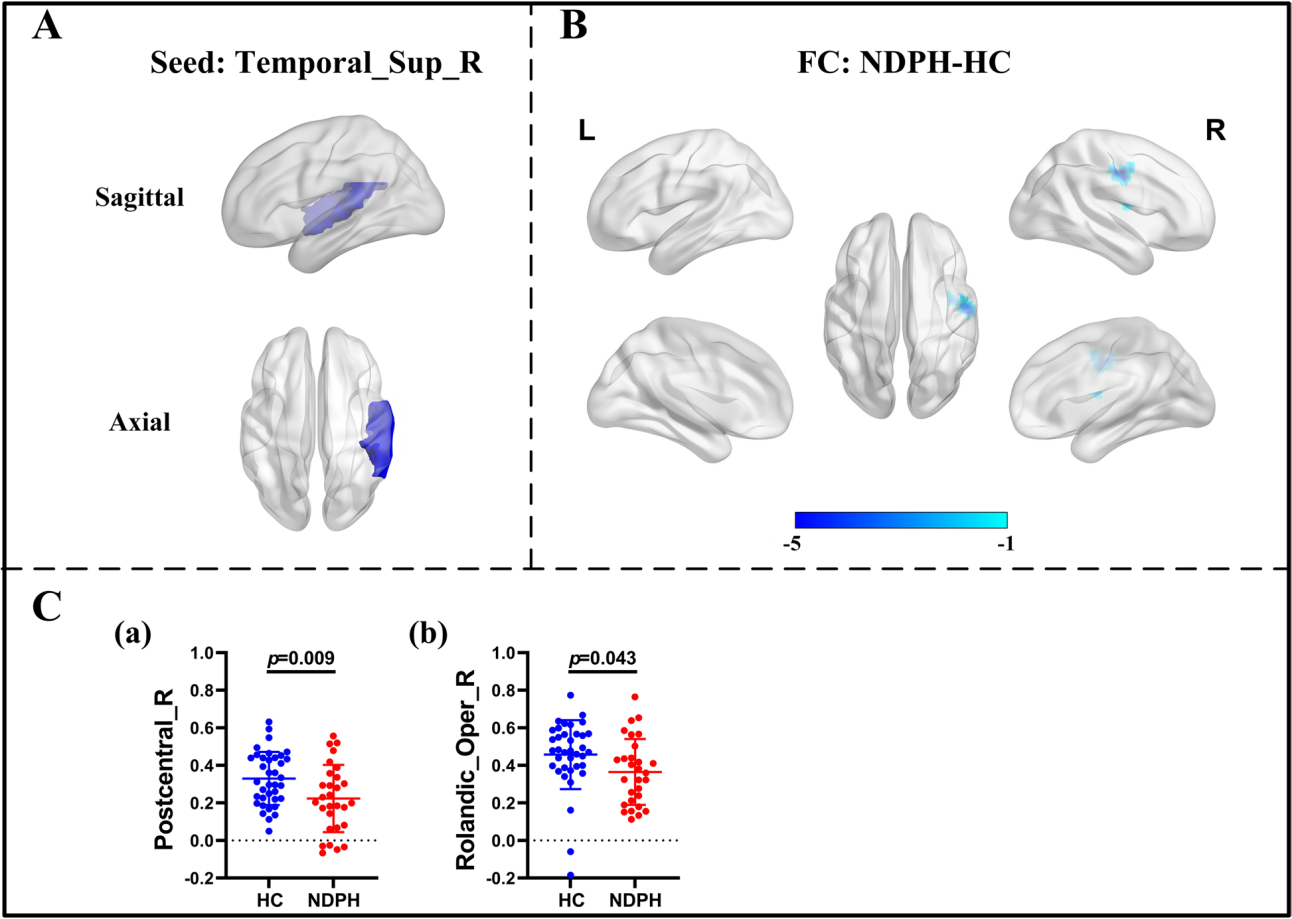
FC analysis from right superior temporal gyrus to the whole brain voxels in NDPH and HC groups. **A** The schematic diagram of the seed (right superior temporal gyrus). The brain region marked in blue represents the right fusiform gyrus. **B** The schematic diagram of significant FC differences. Compared with HCs, patients with NDPH showed significantly increased FC between the seed and right postcentral gyrus, right rolandic operculum. **C** (a) and (d) represented FC values between the seed and right postcentral gyrus, right rolandic operculum respectively in HC group and patients with NDPH. Notes: The color bar showed t-values of the two-sample t-tests on FC. Abbreviations: L, left; R, right; Oper, operculum; FC, functional connectivity; NDPH, new daily persistent headache; HC, health control

Based on 116 brain regions of the AAL atlas, we finally found abnormal FC in 10 seeds including the right lingual gyrus, left superior occipital gyrus, right middle occipital gyrus, bilateral inferior occipital gyrus, right fusiform gyrus, bilateral postcentral gyrus, right thalamus, and right superior temporal gyrus. The results were as follows: (1) The FC between the right lingual gyrus and bilateral calcarine (Fig. 1A–C; L: *p* < 0.001; R:* p* = 0.010), right fusiform gyrus (Fig. 1A–C; *p* = 0.022) were significantly decreased in the NDPH group compared to the HC group. (2) The FC between the left superior occipital gyrus and right lingual gyrus (Fig. 2A; *p* = 0.006), right fusiform gyrus (Fig. 2A; *p* = 0.020) were significantly decreased in the NDPH group compared to the HC group. (3) The FC between the right middle occipital gyrus and bilateral lingual gyrus (Fig. 2B; L: *p* < 0.001; R:* p* < 0.001), right calcarine (Fig. 2B; *p* = 0.002), right fusiform gyrus (Fig. 2B; *p* = 0.007) were significantly decreased in the NDPH group compared to the HC group. (4) Compared with HCs, patients with NDPH showed significantly increased FC between the left inferior occipital gyrus and right superior frontal gyrus (Fig. 2C; *p* = 0.008) and decreased FC between the left inferior occipital gyrus and bilateral lingual gyrus (Fig. 2C; L: *p* = 0.005; R:* p* = 0.023), left fusiform gyrus (Fig. 2C; *p* = 0.010), bilateral inferior occipital gyrus (Fig. 2C; L: *p* = 0.032; R:* p* = 0.012), right middle occipital gyrus (Fig. 2C; *p* = 0.029). (5) The FC between the right inferior occipital gyrus and bilateral lingual gyrus (Fig. 2D; L: *p* = 0.002; R:* p* = 0.003), bilateral fusiform gyrus (Fig. 2D; *p* = 0.029; R:* p* = 0.041), left inferior occipital gyrus (Fig. 2D; *p* = 0.045) were significantly decreased in the NDPH group compared to the HC group. (6) The FC between the right fusiform gyrus and bilateral lingual gyrus (Fig. 3A-C; L: *p* = 0.007; R:* p* = 0.005), right Inferior occipital gyrus (Fig. 3A-C; *p* = 0.020), right calcarine (Fig. 3A-C; *p* = 0.008), right cuneus (Fig. 3A-C; *p* = 0.005), right superior occipital gyrus (Fig. 3A-C; *p* = 0.008) were significantly decreased in the NDPH group compared to the HC group. (7) The FC between the left postcentral gyrus and right precentral gyrus (Fig. 4A; *p* = 0.016), right postcentral gyrus (Fig. 4A; *p* = 0.005) were significantly decreased in the NDPH group compared to the HC group. The FC between the right postcentral gyrus and bilateral precentral gyrus (Fig. 4B; L: *p* = 0.020; R: *p* = 0.015), left postcentral gyrus (Fig. 4B; *p* < 0.001) were significantly decreased in the NDPH group compared to the HC group. (8) The FC between the right thalamus and bilateral cuneus (Fig. 5A-C; L: *p* = 0.015; R: *p* = 0.025), left calcarine (Fig. 5A-C; *p* = 0.031), left superior occipital gyrus (Fig. 5A-C; *p* = 0.042) were significantly increased in the NDPH group compared to the HC group. (9) The FC between the right superior temporal gyrus and right postcentral gyrus (Fig. 6A-C; *p* = 0.009), right rolandic operculum (Fig. 6A-C; *p* = 0.043) were significantly decreased in the NDPH group compared to the HC group. Specific brain regions coordinates and cluster details were listed in Table 2.

**Table 2:** Brain regions with aberrant functional connectivity

Brain regions	Side	MNI coordinates			Peak *t*-valve	Cluster size	Cluster level P_FWE corr_
		x	y	z			
Seed: right lingual gyrus; FC: patients < HCs							
Cluster 1		12	-63	6	3.23	321	< 0.001
Calcarine	R						
Fusiform gyrus	R						
Calcarine	L						
Seed: left superior occipital gyrus; FC: patients < HCs							
Cluster 1		18	-81	0	4.51	118	0.027
Lingual gyrus	R						
Fusiform gyrus	R						
Seed: right middle occipital gyrus; FC: patients < HCs							
Cluster 1		21	-45	3	4.89	481	< 0.001
Lingual gyrus	R						
Lingual gyrus	L						
Calcarine	R						
Fusiform gyrus	R						
Seed: left inferior occipital gyrus; FC: patients > HCs							
Cluster 1		3	45	51	4.47	129	0.021
Superior frontal gyrus, medial	R						
Seed: left inferior occipital gyrus; FC: patients < HCs							
Cluster 1		-30	-78	6	5.12	234	0.001
Lingual gyrus	L						
Fusiform gyrus	L						
Inferior occipital gyrus	L						
Cluster 2		39	-84	0	4.31	182	0.005
Middle occipital gyrus	R						
Lingual gyrus	R						
Inferior occipital gyrus	R						
Seed: right inferior occipital gyrus; FC: patients < HCs							
Cluster 1		-12	-78	12	5.07	298	< 0.001
Lingual gyrus	L						
Fusiform gyrus	L						
Inferior occipital gyrus	L						
Cluster 2		21	-45	3	4.39	225	0.002
Lingual gyrus	R						
Fusiform gyrus	R						
Seed: right fusiform gyrus; FC: patients < HCs							
Cluster 1		27	-66	-9	5.36	786	< 0.001
Lingual gyrus	R						
Lingual gyrus	L						
Inferior occipital gyrus	R						
Calcarine	R						
Cluster 2		15	-81	39	5.05	160	0.007
Cuneus	R						
Superior occipital gyrus	R						
Seed: left postcentral gyrus; FC: patients < HCs							
Cluster 1		45	-18	33	4.28	298	< 0.001
Postcentral gyrus	R						
Precentral gyrus	R						
Seed: right postcentral gyrus; FC: patients < HCs							
Cluster 1		-42	-12	48	4.69	494	< 0.001
Postcentral gyrus	L						
Precentral gyrus	L						
Cluster 2		30	-27	60	4.56	166	0.008
Precentral gyrus	R						
Seed: right thalamus; FC: patients > HCs							
Cluster 1		-6	-90	18	4.64	217	0.004
Cuneus	L						
Cuneus	R						
Calcarine	L						
Superior occipital gyrus	R						
Seed: right superior temporal gyrus; FC: patients < HCs							
Cluster 1		42	0	18	4.59	108	0.035
Postcentral gyrus	R						
Rolandic operculum	R						

*FC* functional connectivity, *HCs* healthy controls, *MNI* Montreal Neurological Institute, *L* left, *R* right, *FWE corr* family wise error correction. Brain region localizations were performed using an automatic anatomical labeling atlas, and the number of voxels of the anatomical regions in which the cluster extends to were reported

### Correlation analysis

In this study, we compared the correlation between seed's FC and patients’ clinical characteristics and neuropsychological tests. There was no significant correlation between the seed's FC and patients’ clinical characteristics, neuropsychological tests, including the disease duration, headache intensity, the score of HIT-6, PHQ-9, GAD-7, PSQI, MoCA after controlling age and sex (Table 3).

**Table 3:** The correlation between FC and clinical characteristics in patients with NDPH

Brain regions	Side	Disease duration *r* (*p*-value)	VAS score *r* (*p*-value)	HIT-6 score *r* (*p*-value)	PHQ-9 score *r* (*p*-value)	GAD-7 score *r* (*p*-value)	PSQI score *r* (*p*-value)	MoCA score *r* (*p*-value)
Seed: right lingual gyrus; FC: patients < HCs								
Cluster 1								
Calcarine	R	0.070 (0.719)	-0.079 (0.686)	0.036 (0.086)	-0.195 (0.339)	0.013 (0.948)	0.256 (0.217)	-0.074 (0.772)
Fusiform gyrus	R	-0.032 (0.870)	0.048 (0.804)	0.120 (0.569)	-0.244 (0.230)	0.114 (0.579)	-0.017 (0.936)	-0.071 (0.780)
Calcarine	L	-0.118 (0.544)	-0.046 (0.814)	-0.137 (0.514)	-0.269 (0.183)	0.014 (0.947)	0.056 (0.792)	0.137 (0.587)
Seed: left superior occipital gyrus; FC: patients < HCs								
Cluster 1								
Lingual gyrus	R	0.205 (0.287)	0.033 (0.865)	0.217 (0.297)	-0.241 (0.236)	0.065 (0.763)	0.185 (0.377)	-0.195 (0.435)
Fusiform gyrus	R	0.039 (0.839)	0.006 (0.977)	0.030 (0.886)	-0.228 (0.263)	0.120 (0.559)	0.015 (0.945)	0.030 (0.905)
Seed: right middle occipital gyrus; FC: patients < HCs								
Cluster 1								
Lingual gyrus	R	0.234 (0.221)	0.116 (0.549)	0.242 (0.243)	-0.277 (0.270)	0.077 (0.710)	0.021 (0.922)	-0.137 (0.586)
Lingual gyrus	L	0.198 (0.303)	0.070 (0.718)	0.276 (0.181)	-0.265 (0.190)	0.081 (0.693)	0.101 (0.630)	-0.069 (0.786)
Calcarine	R	0.185 (0.335)	0.051 (0.792)	0.268 (0.194)	-0.322 (0.109)	0.022 (0.915)	-0.056 (0.790)	-0.008 (0.975)
Fusiform gyrus	R	0.049 (0.803)	0.102 (0.598)	0.057 (0.787)	-0.188 (0.358)	0.160 (0.435)	0.037 (0.862)	0.319 (0.397)
Seed: left inferior occipital gyrus; FC: patients > HCs								
Cluster 1								
Superior frontal gyrus, medial	R	0.023 (0.907)	0.192 (0.319)	0.158 (0.452)	-0.019 (0.925)	0.236 (0.247)	0.093 (0.660)	0.104 (0.681)
Seed: left inferior occipital gyrus; FC: patients < HCs								
Cluster 1								
Lingual gyrus	L	-0.038 (0.845)	-0.010 (0.961)	0.254 (0.220)	-0.257 (0.204)	-0.029 (0.889)	-0.025 (0.907)	-0.209 (0.406)
Fusiform gyrus	L	-0.054 (0.779)	0.143 (0.461)	0.112 (0.594)	-0.184 (0.369)	0.126 (0.540)	0.068 (0.746)	0.032 (0.898)
Inferior occipital gyrus	L	0.017 (0.932)	0.033 (0.864)	-0.009 (0.966)	0.258 (0.203)	0.099 (0.630)	-0.009 (0.967)	-0.084 (0.739)
Cluster 2								
Middle occipital gyrus	R	0.312 (0.100)	0.054 (0.780)	0.225 (0.279)	-0.175 (0.392)	0.131 (0.525)	0.160 (0.444)	-0.144 (0.568)
Lingual gyrus	R	-0.017 (0.931)	0.065 (0.738)	0.207 (0.321)	-0.280 (0.166)	-0.099 (0.631)	0.028 (0.895)	0.106 (0.675)
Inferior occipital gyrus	R	0.104 (0.593)	0.114 (0.555)	-0.111 (0.597)	-0.288 (0.154)	0.028 (0.892)	-0.034 (0.873)	-0.326 (0.186)
Seed: right inferior occipital gyrus; FC: patients < HCs								
Cluster 1								
Lingual gyrus	L	0.046 (0.813)	0.010 (0.958)	0.297 (0.150)	0.246 (0.226)	0.017 (0.936)	-0.124 (0.555)	0.053 (0.833)
Fusiform gyrus	L	0.050 (0.797)	0.003 (0.986)	0.125 (0.552)	0.254 (0.210)	0.049 (0.812)	0.151 (0.471)	0.124 (0.624)
Inferior occipital gyrus	L	0.069 (0.721)	0.077 (0.691)	-0.051 (0.807)	0.265 (0.191)	0.027 (0.894)	0.027 (0.897)	0.184 (0.464)
Cluster 2								
Lingual gyrus	R	0.058 (0.765)	0.067 (0.730)	0.231 (0.266)	0.265 (0.191)	0.059 (0.773)	0.111 (0.596)	-0.078 (0.759)
Fusiform gyrus	R	-0.093 (0.633)	0.130 (0.500)	0.142 (0.499)	-0.186 (0.364)	0.080 (0.698)	0.031 (0.884)	0.126 (0.618)
Seed: right fusiform gyrus; FC: patients < HCs								
Cluster 1								
Lingual gyrus	R	0.092 (0.634)	0.106 (0.586)	0.234 (0.261)	-0.190 (0.354)	0.069 (0.737)	0.126 (0.548)	-0.105 (0.679)
Lingual gyrus	L	0.088 (0.648)	0.013 (0.947)	0.204 (0.328)	-0.219 (0.283)	0.084 (0.684)	0.083 (0.695)	-0.018 (0.943)
Inferior occipital gyrus	R	0.054 (0.782)	0.153 (0.429)	0.115 (0.584)	-0.204 (0.317)	0.014 (0.944)	-0.012 (0.956)	-0.315 (0.203)
Calcarine	R	0.077 (0.690)	-0.004 (0.982)	0.283 (0.171)	0.189 (0.354)	0.017 (0.934)	0.134 (0.524)	-0.102 (0.687)
Cluster 2								
Cuneus	R	0.245 (0.201)	-0.002 (0.992)	0.286 (0.165)	-0.245 (0.227)	0.047 (0.819)	0.005 (0.981)	0.034 (0.894)
Superior occipital gyrus	R	0.156 (0.420)	-0.166 (0.510)	0.213 (0.306)	-0.15 (0.465)	0.075 (0.718)	0.144 (0.492)	-0.166 (0.510)
Seed: left postcentral gyrus; FC: patients < HCs								
Cluster 1								
Postcentral gyrus	R	0.176 (0.361)	0.286 (0.132)	0.042 (0.842)	-0.134 (0.514)	0.211 (0.300)	-0.009 (0.967)	0.074 (0.771)
Precentral gyrus	R	-0.010 (0.959)	0.232 (0.225)	0.051 (0.810)	-0.154 (0.453)	0.081 (0.695)	-0.031 (0.885)	0.090 (0.722)
Seed: right postcentral gyrus; FC: patients < HCs								
Cluster 1								
Postcentral gyrus	L	0.113 (0.559)	0.178 (0.355)	0.002 (0.991)	0.226 (0.267)	0.141 (0.492)	-0.069 (0.742)	-0.078 (0.757)
Precentral gyrus	L	-0.011 (0.956)	0.176 (0.362)	0.021 (0.920)	-0.213 (0.296)	-0.081 (0.695)	-0.113 (0.692)	0.189 (0.452)
Cluster 2								
Precentral gyrus	R	-0.092 (0.635)	0.225 (0.231)	-0.072 (0.731)	-0.171 (0.404)	0.016 (0.939)	0.201 (0.425)	0.229 (0.231)
Seed: right thalamus; FC: patients > HCs								
Cluster 1								
Cuneus	L	-0.173 (0.370)	-0.062 (0.750)	0.208 (0.319)	0.237 (0.243)	0.118 (0.566)	-0.043 (0.839)	-0.015 (0.952)
Cuneus	R	-0.136 (0.483)	-0.071 (0.713)	0.166 (0.427)	0.232 (0.255)	0.075 (0.716)	-0.055 (0.794)	-0.087 (0.731)
Calcarine	L	0.183 (0.343)	0.062 (0.751)	0.195 (0.349)	-0.107 (0.602)	0.157 (0.445)	0.008 (0.971)	0.023 (0.928)
Superior occipital gyrus	R	-0.018 (0.924)	0.121 (0.531)	0.330 (0.107)	0.176 (0.389)	0.122 (0.554)	0.014 (0.949)	-0.231 (0.356)
Seed: right superior temporal gyrus; FC: patients < HCs								
Cluster 1								
Postcentral gyrus	R	-0.074 (0.704)	0.289 (0.129)	0.252 (0.223)	0.018 (0.932)	0.183 (0.370)	0.042 (0.842)	0.339 (0.169)
Rolandic operculum	R	0.042 (0.828)	0.371 (0.048)	0.350 (0.087)	-0.083 (0.688)	0.088 (0.688)	0.220 (0.290)	0.220 (0.380)

*Abbreviations*: *HCs* healthy controls, *VAS* visual analogue scale, *HIT-6* headache impact test, *PHQ-9* patient health questionnaire-9, *GAD-7* generalized anxiety disorder-7, *PSQI* Pittsburgh sleep quality index, *MoCA* Montreal cognitive assessment. Statistical significance: *p* < 0.00019 (Bonferroni correction)

## Discussion

In this study, we defined 116 brain regions in the AAL atlas as seeds to map the aberrant FC and aimed to elucidate the functional network involved in the pathogenesis of NDPH. Our results showed abnormal FC in multiple brain regions involved in the perception and regulation of emotion and pain.

### Aberrant FC of the occipital lobe in headache and pain

In this study, we found abnormal FC in multiple seeds (including lingual gyrus, superior occipital gyrus, middle occipital gyrus, and inferior occipital gyrus) of the occipital lobe in patients with NDPH. The occipital lobe is the visual processing center of the mammalian brain containing most of the anatomical region of the visual cortex [18]. There was some evidence that structural and functional abnormalities of the occipital lobe were involved in the pathology of headache and pain. The magnetic resonance spectroscopy (MRS) studies provided evidence that migraine patients have altered excitability of the brain as well as abnormal levels of the neurotransmitters glutamate, γ-aminobutyric acid (GABA), glutathione, choline, and phosphates in the occipital cortex involved in pain processing [19–24]. Animal models showed that the trigeminal nucleus caudalis projects indirectly to primary visual cortex involved in the affective/motivational aspects of the pain perception [25]. Additionally, the photophobia that accompanies the migraine attack has been modeled in animals, the primary and secondary visual cortex are involved in photophobia circuits in migraine-relevant pain [25]. A review summarizes the abnormal FC in multiple subregions of the occipital lobe in migraine patients, and it suggests that the abnormal functional network of the occipital lobe is involved in headache regulation [26]. Some studies suggested that migraine with visual aura exist in altered neurochemical coupling in the occipital cortex [19]. Our recent study also reported decreased cerebral perfusion, increased regional homogeneity and multiple frequency amplitudes of low-frequency fluctuation in the middle occipital gyrus in patients with NDPH [8, 27]. The above evidence suggests that the abnormal functional network and imbalance of neurotransmitters of the occipital lobe might be involved in headache and pain. Our results showed significantly reduced FC between multiple subregions of the occipital lobe in patients with NDPH and we preliminarily concluded that the inherent abnormal FC in the occipital lobe might be involved in the pathological mechanism of pain and photophobia on NDPH.

### Aberrant FC of the fusiform gyrus in headache and pain

The fusiform gyrus is an integrative zone participating in the mental imagery process related to the retrieval of pain perception [28]. Previous studies have shown that abnormal function and structure of the fusiform gyrus can exist in patients with chronic low back pain, fibromyalgia, migraine, and cluster headache [29–32]. There was a view that the fusiform gyrus as a cognitive pain-processing region might be involved in the regulation of headaches and pain [33]. Some evidence also suggests that in psychosis, the fusiform gyrus is involved in emotional recognition and emotional intensity recognition [34, 35]. In this study, patients with NDPH had a higher proportion of anxiety, depression, and other emotional disorders which might be involved in emotional cognitive regulation of the fusiform gyrus. Additionally, we found that compared with HCs, the NDPH group had significantly decreased FC between the fusiform gyrus and multiple subregions of the occipital lobe. In summary, we speculate that abnormal functional connections between the fusiform gyrus and occipital lobe are involved in pain processing and emotional regulation in patients with NDPH.

### Aberrant FC of the superior temporal gyrus in headache and pain

The superior temporal gyrus has been involved in auditory processing, including language, and perception of emotion in facial stimuli, but also has been implicated as a critical structure in social cognition [36–38]. Recent neuroimage studies have shown that alterations in the structure and function of the superior temporal gyrus can be used as possible biomarkers of psychosis and psychological disorders [39, 40]. In the field of headache, some studies have shown that enhanced regional functional activity of the superior temporal gyrus in patients with vestibular migraine is presumed to be related to auditory information processing [41], abnormal neurotransmitters levels of temporal lobe existed in patients with migraine [21] and the structural alterations of superior temporal gyrus identified as a relevant factor to differentiate adult patients with chronic migraine from those with episodic migraine and HCs [42]. In our study, more than half of patients were accompanied by phonophobia, 44.0% of patients had depression and 32.0% of patients had generalized anxiety disorder. Our results showed that compared to HCs, patients with NDPH had significantly reduced FC between the superior temporal gyrus and the sensory cortex. This also confirms previous studies that reduced cortical thickness of the superior temporal gyrus in adolescents with NDPH [7] and decreased cerebral perfusion of temporal gyrus in adult patients with NDPH [8]. In summary, our results demonstrated the dysfunction of superior temporal gyrus might be mainly involved in emotion regulation, auditory information processing in patients with NDPH.

### Aberrant FC of the thalamus in headache and pain

As the most giant gray matter nucleus in the diencephalon, the thalamus is the core of the central integration and processing of nociceptive signals [43]. Our results illustrated significantly increased FC between the thalamus and multiple subregions of the occipital lobe. Studies have shown that 33–69% of patients with NDPH suffer from photophobia [1]. In this study, 37.93% of patients with NDPH had accompanying photophobia. Studies in rats have shown that posterior thalamic neurons were specifically activated by light, and this activation increases with increasing light intensity [44]. Ganglion cells project visual signals to a subgroup of posterior thalamic neurons and these neurons continue to project to visual, somatosensory, and associative cortices. Previous studies have suggested that abnormal visual cortex activation and thickness might be the pathological mechanism of photophobia in patients with headaches [45–48]. The traditional pathway of vision is as follows [49]: ganglion cell neurons transmit visual signals generated by the retina to the optic nerve. The optic nerve transmits these optical signals to the lateral geniculate nucleus of the thalamus. Finally, the lateral geniculate nucleus continues to project visual cues onto the primary and secondary visual cortices. Our results showed significantly enhanced FC between the thalamus and visual cortex (the cuneus, calcarine, and superior occipital lobe). This result further validates the previous photophobic mechanism of headaches. Therefore, we conclude that dysfunction of the thalamus-visual cortex pathway might be one of the mechanisms of photophobia in patients with NDPH and might be involved in pain processing.

### Aberrant FC of the postcentral gyrus in headache and pain

The postcentral gyrus forms the primary somatosensory cortex and is referred to as Brodmann area 1, 2 and 3. This gyrus receives sensory information from all the sensory receptors that provide information related to temperature, pain (spinothalamic pathway), vibration, proprioception, and fine touch [42]. A magnetoencephalography study showed that the somatosensory cortex's activity rises with both the intensity of nociceptive stimulation and subjective pain evaluations [50]. It confirmed that the somatosensory cortex not only contributes to sensorimotor integration but is also involved in processing pain. Our results showed that patients with NDPH had significantly reduced FC within the sensory cortex. This might be the underlying mechanisms of persistent headache due to the decreased pain integration ability of the sensory cortex.

#### Limitations

This study has some limitations. First, this study is a single-center study with a small sample size, and our preliminary results need to be verified with more data. Second, attributed to a cross-sectional study, we can not trace the change of FC during disease progression and/or whether they are reversible. Longitudinal studies are necessary to evaluate the correlation between FC and prognosis and disease progression. Additionally, we used the 116 brain regions AAL atlas as seeds in this study and did not analyze the subregions of these seeds. However, different subregions of seeds might have different physiological functions, which might affect the analyses of FC. Finally, since psychiatric comorbidity and in particular chronic anxiety and depression, even if low-grade and untreated, could affect the FC of NDPH, further studies are needed to determine whether this type of comorbidity may have contributed to the FC abnormalities detected here. Therefore, it is necessary further to explore the FC in different subregions of these seeds to better understand the central pathogenesis of NDPH.

## Conclusions

Considering the presence of persistent pain in NDPH, lack of an identifiable cause, and absence of evidence-based medical evidence and effective treatment, there is an urgent need to explore this highly disabling but low-incidence disease. This is a study exploring the FC of multiple brain regions with NDPH in adults. Our findings suggest that patients with NDPH have abnormal FC in multiple brain regions involved in the perception and regulation of emotion and pain. Moreover, this study provides evidence of central nervous system dysfunction to understand the pathogenesis of associated symptoms in NDPH and provides a new way for physicians to seek approaches to terminate chronic pain.
